# Populating the Data Ark: An attempt to retrieve, preserve, and liberate data from the most highly-cited psychology and psychiatry articles

**DOI:** 10.1371/journal.pone.0201856

**Published:** 2018-08-02

**Authors:** Tom E. Hardwicke, John P. A. Ioannidis

**Affiliations:** 1 Meta-Research Innovation Center at Stanford (METRICS), Stanford University, Stanford, California, United States of America; 2 Departments of Medicine, of Health Research and Policy, of Biomedical Data Science, and of Statistics, Stanford University, Stanford, California, United States of America; Tilburg University, NETHERLANDS

## Abstract

The vast majority of scientific articles published to-date have not been accompanied by concomitant publication of the underlying research data upon which they are based. This state of affairs precludes the routine re-use and re-analysis of research data, undermining the efficiency of the scientific enterprise, and compromising the credibility of claims that cannot be independently verified. It may be especially important to make data available for the most influential studies that have provided a foundation for subsequent research and theory development. Therefore, we launched an initiative—the Data Ark—to examine whether we could retrospectively enhance the preservation and accessibility of important scientific data. Here we report the outcome of our efforts to retrieve, preserve, and liberate data from 111 of the most highly-cited articles published in psychology and psychiatry between 2006–2011 (n = 48) and 2014–2016 (n = 63). Most data sets were not made available (76/111, 68%, 95% CI [60, 77]), some were only made available with restrictions (20/111, 18%, 95% CI [10, 27]), and few were made available in a completely unrestricted form (15/111, 14%, 95% CI [5, 22]). Where extant data sharing systems were in place, they usually (17/22, 77%, 95% CI [54, 91]) did not allow unrestricted access. Authors reported several barriers to data sharing, including issues related to data ownership and ethical concerns. The Data Ark initiative could help preserve and liberate important scientific data, surface barriers to data sharing, and advance community discussions on data stewardship.

## Introduction

Scientific claims are frequently made without concomitant publication of the underlying research data upon which they are based [1,2]. Post-publication efforts to obtain data directly from study authors also tend to be unsuccessful in the majority of cases [3–5]. This state of affairs precludes the routine re-use and re-analysis of scientific data, undermining the efficiency and credibility of the scientific enterprise [6–8].

Several initiatives are trying to increase the availability of raw data from scientific studies. Some journals in diverse fields such as genetics, statistics, and clinical research have already adopted policies that require public availability of raw data as a prerequisite to publication for specific types of investigation [9,10]. But even for journals with relatively stringent data sharing policies, compliance can vary considerably in practice [11–15]. Regardless, stringent data sharing policies only account for a minority of journals and the vast majority of research data is not readily available [1,2,10].

A series of new campaigns are trying to strengthen data sharing policies at institutions, funders, and journals [16], incentivize voluntary sharing upon publication with a ‘badges’ system [17], and promote a grassroots effort by peer-reviewers to mandate inclusion of explicit data availability statements in research reports [18]. Crucially, all of these initiatives are directed at improving the data sharing landscape prospectively. They do not address the fact that the data from most already published studies is likely to be unavailable. As a result, the majority of the extant scientific literature may not be directly verifiable by independent researchers: a feature often considered a hallmark of the scientific endeavour [19,20].

Recently, we launched an initiative that attempts to retrospectively retrieve, preserve, and liberate important scientific data. Our intention was to create an online data repository—the Data Ark (https://osf.io/view/DataArk/)—and populate it with data sets, enabling the research community to re-use and verify them. Trying to retrieve all the raw data from past studies is likely to be very difficult and utopian. If one were to prioritize, it would seem most important to retrieve and preserve data from the studies that have been relied upon most frequently in subsequent research and theory development. It may be of questionable value to retrieve, clean, and share the data from an abandoned study that has received little attention from the scientific community. Thus, we initially prioritized studies that have been cited extensively in the literature.

Here we report the outcome of our initial efforts to retrieve data from 111 of the most highly-cited articles published in psychology and psychiatry between 2006 and 2016. We contacted research teams and suggested three sharing options: (A) sharing in the Data Ark with unrestricted access; (B) sharing in the Data Ark with restricted access (in which case, we asked the researchers to specify the required restrictions); or (C) sharing with our research team (the Meta-Research Innovation Center at Stanford [METRICS]) only. If researchers were unable or unwilling to share, we asked them to specify the key reasons so that we could learn more about potential barriers to data sharing. If a data sharing system was already in place, we recorded any restrictions we encountered when attempting to obtain the relevant data set.

## Results

Our eligible sample comprised of the 48 most-highly-cited studies with primary data in psychology and psychiatry published between 2006 and 2011 (median 560 citations by 31st May, 2017, range 423 to 1768 citations) and the 63 most highly-cited studies with primary data in psychology and psychiatry published between 2014 and 2016 (median 57 citations by 31st May, 2017, range 45 to 147 citations). These studies are among the top 0.11% and top 0.16% of citations for 2006–2011 and 2014–2016, respectively.

**Fig 1:**
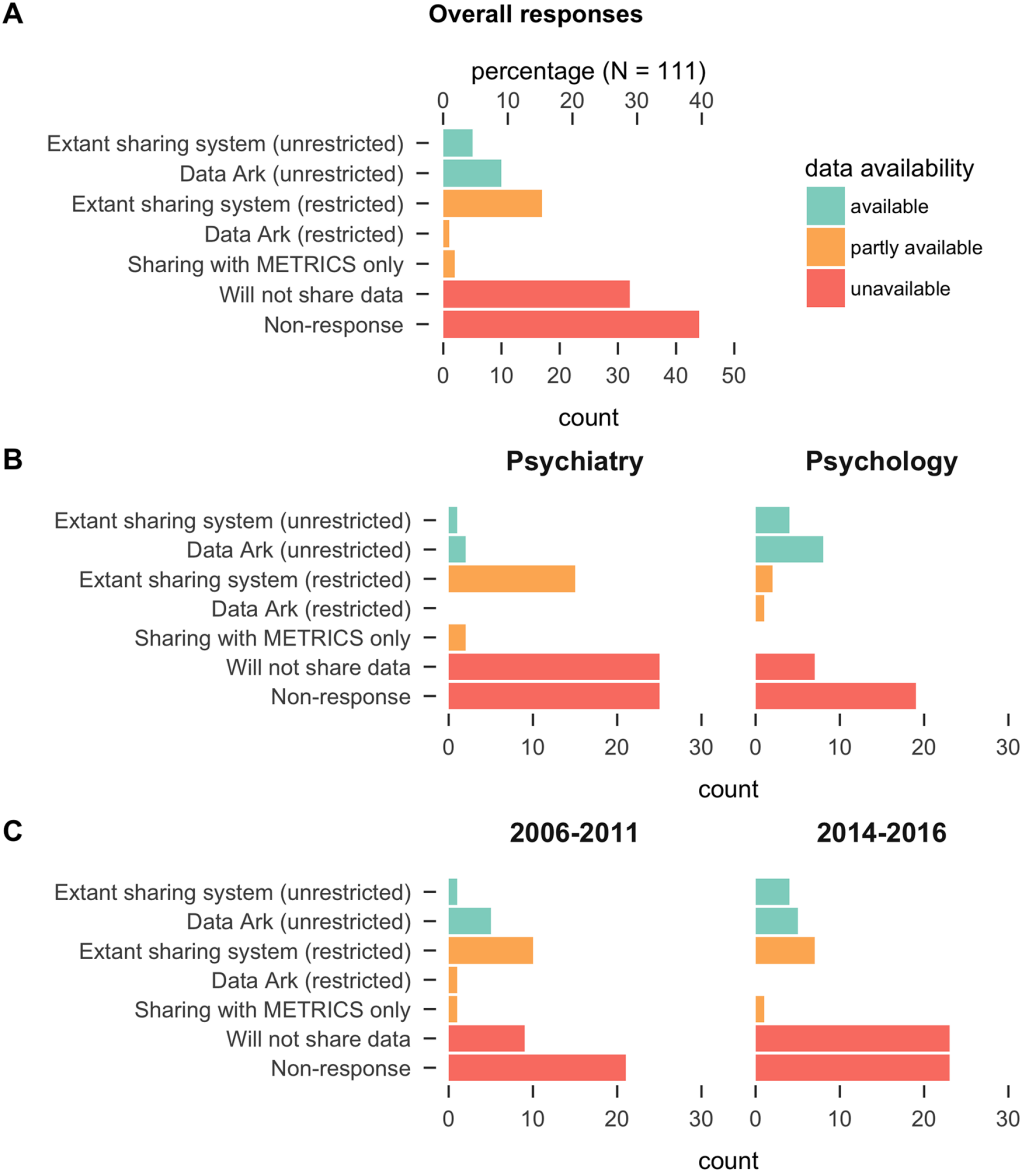
Responses to data request, overall (Panel A) and broken down by field (Panel B) and sample time period (Panel C).

Response outcomes are displayed in Fig 1, overall (Panel A) and broken down by either field (Panel B) or time period (Panel C). Values in square brackets are 95% confidence intervals (CIs) based on the Wilson method with continuity correction for binomial proportions [21] and the Sison-Glaz method for multinomial proportions [22]. 80 (72%) researchers responded to our initial data request (response time median 7.50 days, range 0 to 35 days). However, 13 of these responses indicated either that data was being located/prepared or our request was being considered: issues that had not been resolved 6 months after our initial contact. As a result, we combined these “non-responses” with the “no response” category.

Overall, data was available with no restrictions for 15 studies (14%, 95% CI [5, 22]), partly available with some restrictions for 20 studies (18%, 95% CI [10, 27]), and unavailable for 76 studies (68%, 95% CI [60, 77]). Of the 15 studies where the data were available with no restrictions, 5 were already made available via an extant data sharing system and 10 were made available to us directly when we prompted the authors to contribute to the Data Ark. These 10 data sets are now available with no restrictions in the Data Ark repository (https://osf.io/view/DataArk/). Of the 20 studies where the data were partly available with some restrictions, 17 were reportedly already available via an extant restricted sharing system (we did not attempt to verify access), 2 were made available only for use by our research team, and 1 was made available for the Data Ark but only if access restrictions could be put in place. There was no case where the data were already available with restrictions and our prompting of the authors resulted in waiver of the restrictions.

The pattern of results was broadly similar across psychology and psychiatry and across the early and late sampling periods (most-cited papers published in 2006–2011 or published in 2014–2016, Fig 1, Panel B and Panel C). Overall, somewhat more data sets were unavailable in the psychology early period (17/24, 71%, 95% CI [49, 87]) relative to the psychology late period (9/17, 53%, 95% CI [29, 76]), but CIs broadly overlapped. The opposite trend was observed for psychiatry: there were somewhat fewer data sets unavailable in the early period (13/24, 54%, 95% CI [33, 74]) relative to the late period (37/46, 80%, 95% CI [66, 90]), but again with broadly overlapping CIs. The proportion of articles with unavailable data exceeded 50% for all types of studies, with the lowest proportion seen for field studies (15/26, 58%, 95% CI [42, 79]) and highest proportion seen for case-control studies (15/16, 94%, 95% CI [88, 100]).

**Fig 2:**
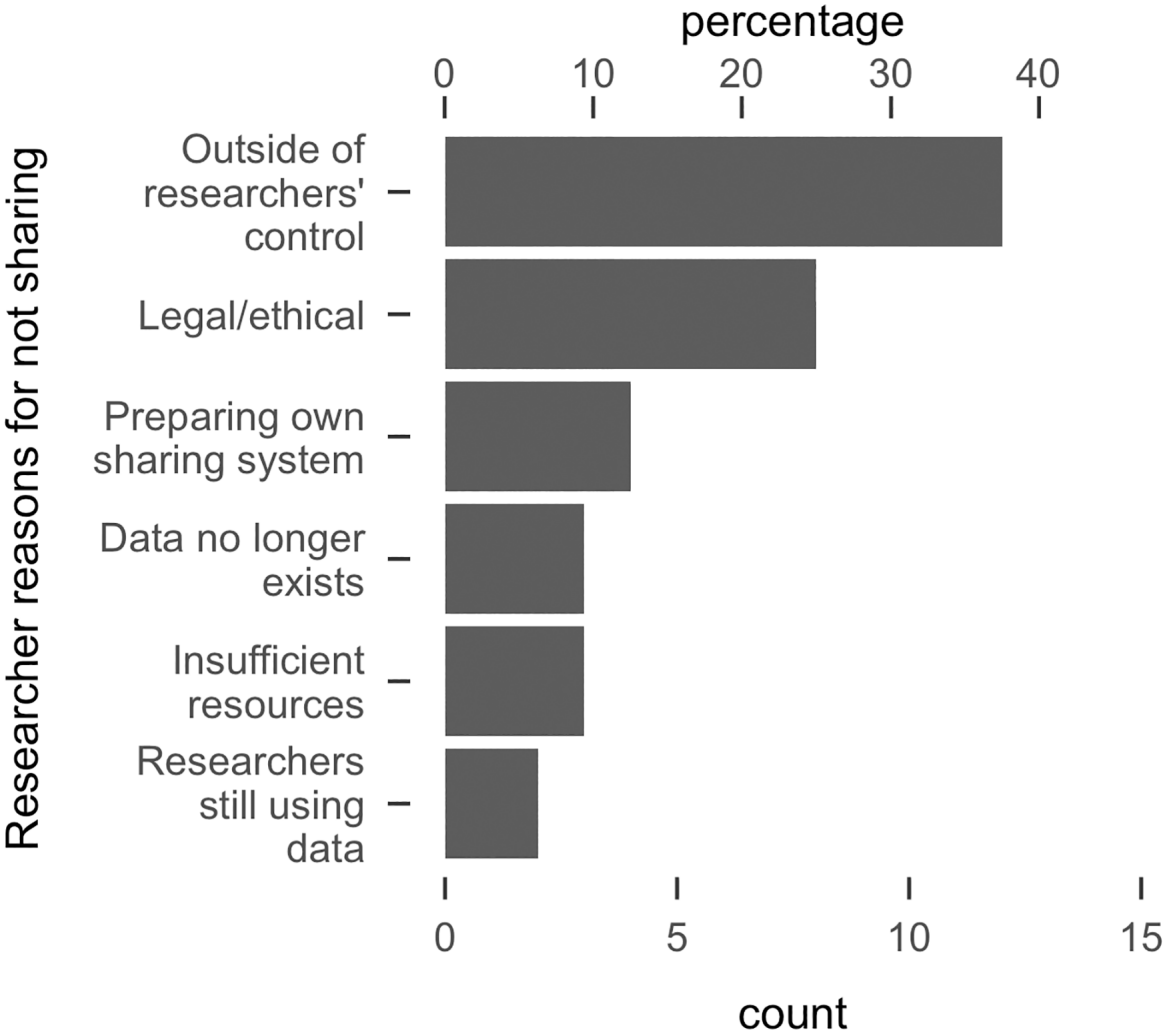
Reasons provided by researchers for not sharing. X-axes represent counts and percentages (of n = 32 who responded that they would not share).

The researcher-provided reasons for not sharing are displayed in Fig 2. In many cases researchers said that data was outside of their control (n = 12) because it had been generated by other researchers (n = 5), or because it was proprietary (n = 7). Several researchers cited legal or ethical concerns (n = 8), for example not explicitly obtaining consent for sharing from study participants (2), laws restricting data access (n = 4), or a lack of approval from their local ethics board (n = 2). A few researchers indicated that they were unwilling to share data with us as they were preparing a data sharing system of their own (n = 4). Some researchers said they would not share right now as they were still using the data, but might share at some (unspecified) time in the future (n = 2). A few researchers said that the data would take time to prepare and they lacked the resources to share (n = 3). Finally, some data had been lost (n = 2), or deliberately destroyed due to privacy concerns (n = 1).

**Fig 3:**
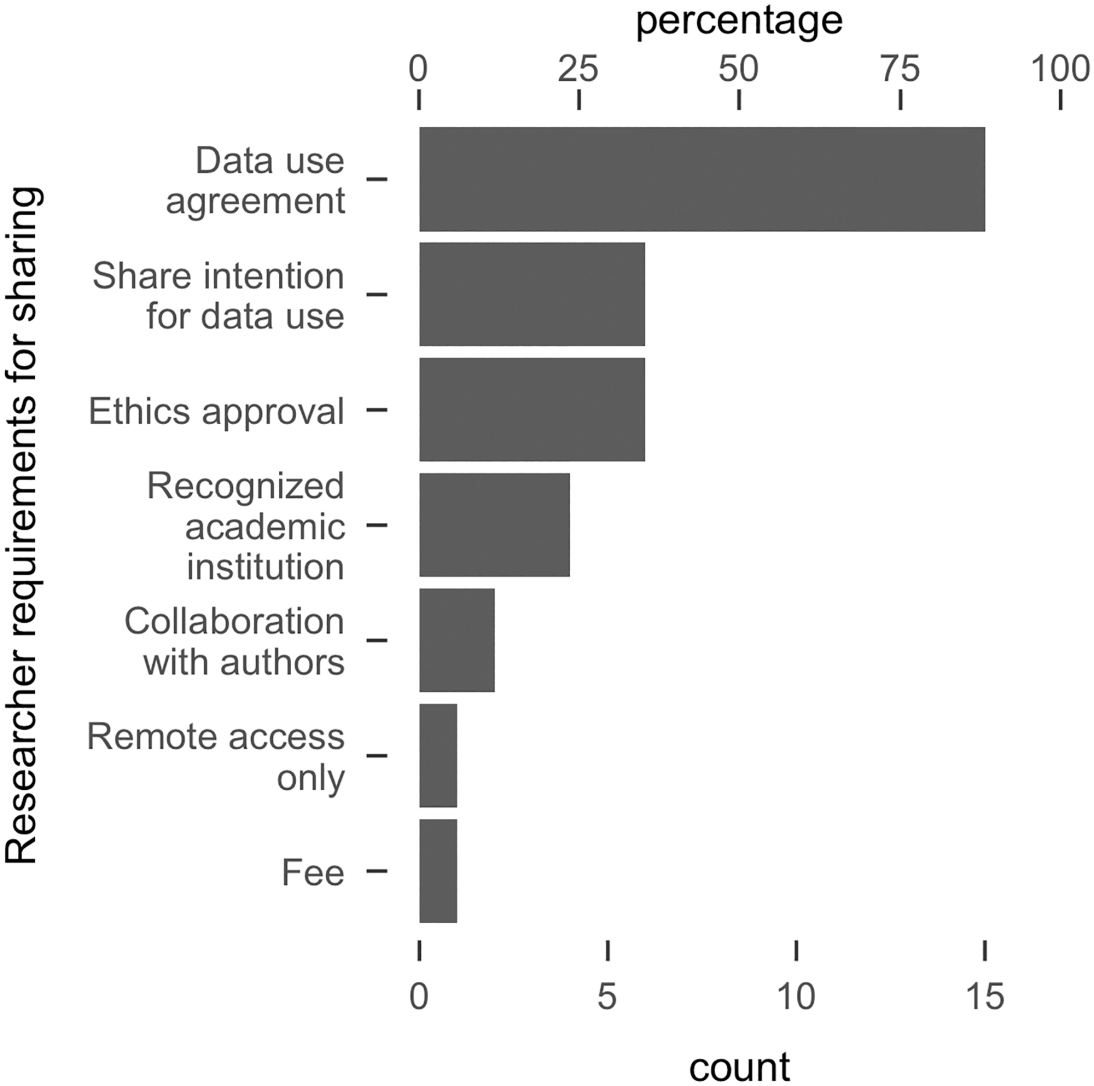
Requirements imposed by researchers before data can be shared. X-axes represent counts and percentages (of n = 17 with restricted data sharing systems in place). Note that these data are not mutually exclusive: individual sharing schemes often entailed multiple restrictions.

Some researchers already had data sharing systems in place (n = 22), but in most cases access was restricted (n = 17, 77%, 95% CI [54, 91]). We did not attempt to access this data but recorded the nature of the restrictions (Fig 3). The vast majority of existing sharing schemes required that we complete a ‘data use agreement’ (n = 15). Generally, these involved pledging not to attempt to identify individual participants, not to independently redistribute the data, store the data securely, and cite the original source in cases of data re-use. Some schemes required data requestors to share their exact intentions for data re-use, for example by detailing any analysis procedures that would be employed (n = 6). It was suggested that such intentions would be vetted and the request would be denied if the analysis was plan was deemed inappropriate or overlapped with some existing research being run by the original research team. Some schemes required that data requestors provided evidence of ethics approval (n = 6) and were employed by a recognized academic institution (n = 4). Other requirements included mandatory collaboration with the original researchers (n = 2), payment of a fee (n = 1), or analysis of the data via a remote access system only (n = 1).

## Discussion

Research data are the core that substantiates scientific claims, but most research data are unavailable [1,2]. Consequently, the evidence underlying most scientific claims is neither re-usable nor verifiable, undermining the efficiency and credibility of the scientific enterprise [6–8]. A number of efforts are underway to improve this situation by requiring or encouraging researchers to make their data available in future studies [16–18]. However, even if these efforts were successful, they would not address the lack of data availability in the extant published literature, and the unavailability of data would be felt most for the studies that have had the greatest influence.

Recently, we launched an initiative to create the Data Ark: an online repository for the preservation of data from already published studies. We initially prioritized studies that have been cited extensively in the literature. Although citations have limitations, they provide an approximation of influence in the scientific literature and are a very widely used measure of impact. Here we have reported the outcomes of our attempts to retrieve, preserve, and liberate the data of the most highly-cited articles published in psychology and psychiatry between 2006 and 2016. The vast majority of data sets were not available (68%), 18% were only partly available with restrictions, and 14% were openly available in a completely unrestricted form. Prompting the authors of these influential studies to contribute their data to the Data Ark repository was not very effective in promoting further sharing: 5 articles already had unrestricted sharing systems in place, and an additional 10 data sets were provided for unrestricted access via the Data Ark.

Previous efforts to obtain data directly from authors ‘upon request’ have also encountered low availability rates; for example, data was available for only 7 out of 157 (4.5%) articles published in the BMJ [11], 48 out of 394 (38%) articles published in four American Psychological Association (APA) journals [4], and 38 out of 141 (27%) articles published in four other APA journals [5]. The highest retrieval rate, 17 out of 37 (46%) articles, was observed for a study focused on data from randomized clinical trials (RCTs) published in the BMJ and PLOS Medicine that both mandate data sharing for RCTs [13]. Thus, even under strict policy requirements, raw data could not be obtained from slightly more than half of the studies. One study [3] suggested that access to research data is being lost at a rate of 17% per year. Our data are too sparsely distributed across publication years to attempt a similar analysis; however, we observed no evidence that the situation has improved in recent years. Our study differed from all previous attempts to retrieve data in that we focused on the most highly-cited articles. One might have hoped that for articles that exert such a major influence in the subsequent literature, their data would have been easier to retrieve and wider availability of this information would complement the legacy of these important works. Nevertheless, our retrieval rates were relatively low.

The pattern of results was broadly similar across the two fields of psychiatry and psychology, and the early and late sampling periods. We cannot exclude the possibility that the proportion of unavailable data may be decreasing, in particular in psychology, but our data were not sufficient to evaluate modest changes in time trends of data availability. The recent impetus for enhancing transparency and reproducibility in psychology [8,18] may lead to greater sharing and this needs to be monitored. It is unknown whether more substantial improvements may be seen in the future in psychology and/or other fields with similarly heightened attention to reproducibility and open science issues.

Data sharing is not always straightforward [23–25] and in some cases the authors’ ability to share may be constrained by legal or ethical obligations. One aim of our project was to surface information about the barriers researchers encounter that can complicate sharing. Interpretation of these responses requires considerable caution because they rely solely on self-report in a context that is likely to be heavily influenced by social desirability. For example, it is perhaps surprising that relatively few respondents to our request indicated that data could not be shared due to limited resources (e.g., insufficient time) as this reasoning is prevalent in anonymous surveys of data sharing attitudes [24]. Additionally, the reasons that influenced non-respondents are unknown and it is possible that despite our best efforts, our request was never read. However, the mere fact that we were unable to elicit a response to our data request from non-respondents suggests that they are more likely to have a less favourable attitude towards data sharing than respondents.

With these caveats in mind, responses appear to suggest that a key barrier to sharing is that data can be outside of an authors’ control, either because the data was generated by other researchers, and sometimes because the data are owned by a commercial entity. This raises important questions about the responsibilities of data stewardship and the ability to verify data that underlies scientific publications. A number of legal/ethical issues were raised, largely relating to the rights and privacy of participants, some of which may be easily resolvable in future studies (e.g., acquiring consent for sharing from participants) and some of which are less straightforward to resolve (e.g., robust de-identification).

A number of researchers already had data sharing systems in place, however many involved restrictions of some kind. Some restrictions were stringent, such as requiring that data requestors submit their intentions for data re-use to a vote of approval by a panel of original research team members or acquire a research position at the original researchers’ institution in order to actively collaborate with them in person. Other restrictions were more straightforward, such as signing a data use agreement. However, it was clear that most restrictions were imposed on an ad-hoc basis by the original authors themselves, again raising important questions about who is the most impartial steward of scientific data. In cases where data cannot be made publicly available, it may be desirable to entrust data to an independent arbitrator who can adjudicate access requests, ensuring that ethical and legal obligations are upheld, whilst maintaining maximal access to data whenever possible [26].

Some limitations of our study need to be discussed. First, the results should not be extrapolated outside the two disciplines that we evaluated. Some scientific disciplines may have a stronger tradition of routine data sharing. For example, data repositories on gene-phenotype associations or microarray data are already very rich, and several other fields in economics, high energy and particle physics, astronomy/astrophysics, and climate change have strong traditions in data sharing. Conversely, other fields have no such tradition and even resistance to data-sharing. Second, it is possible that sharing might have been more likely to occur if we had approached the investigators with a specific request for a scientific project in which they could participate and potentially co-author the resulting work. For example, a recent effort was successful in acquiring data underlying many important findings in the field of memory research in order to establish an empirical common ground for the purposes of computational modeling[27]. By contrast, a request to populate the Data Ark may not be seen as a high priority or personally rewarding. Third, we did not offer any incentives to the authors to share their data. It is unknown if some financial or other incentive would have promoted further sharing. A culture change may nevertheless be possible and there are also other initiatives that aim to promote sharing and distributed collaboration (e.g., [28]). Fourth, we did not attempt to evaluate the quality of data that were contributed to us. Nor we did we attempt to re-analyse it ourselves. Just because data is available, does not guarantee that is re-usable, or that the findings based upon it are reproducible [12,13,15,17].

In summary, we were unable to retrieve data from the majority of the most highly-cited studies published between 2006 and 2016 in the fields of psychology and psychiatry. This means that even though these studies are some of the most influential in these fields and many other scientists have used them extensively, their claims have to be accepted based on trust and the data in question are no longer independently verifiable, or freely re-usable. We acknowledge that data sharing is not always straightforward, and in some cases it will be reasonable to restrict access to sensitive information. However, we believe that higher levels of sharing should be attained. This is even more important for studies that have a major influence on the rest of the scientific literature. While retrieval of all past data would require enormous resources and may meet with justifiable resistance, we argue that for the top of the most-cited literature there should be greater consensus that these data are essential to salvage and make widely accessible to the scientific community.

The demise of the Great Library of Alexandria resulted in the catastrophic destruction of innumerable ancient works. Yet the gradual attrition of scientific data in the modern era is widely tolerated [19]. The modern scholarly record is not under threat from a great fire or flood, but action is needed to ensure that important data does not slip away beyond retrieval [1–5,11–15]. Storage in a Data Ark, an independent repository that will continue to float above the murky depths, may enable long-term preservation and accessibility of the evidence that underlies the scientific literature. The findings reported here suggest that retrospectively retrieving data from highly cited studies is challenging. It may be that most of the data generated by humanity’s previous scientific endeavors is now irrecoverably lost. Nevertheless, growing awareness of the importance of data sharing and research transparency across the sciences is encouraging. As open science becomes normalized and the benefits of data sharing are realized, authors may become more amenable to releasing data from previous studies. We suspect that scientists attempting to retrieve data within their own research communities, perhaps in the service of a defined scientific project, may be able to make a more compelling case to their colleagues that data sharing is worthwhile. Therefore, we propose that researchers who value the preservation of important scientific data take ownership of the process and attempt to establish Data Arks of their own. As indicated by the present results, even if the data yield is not high, it is useful to surface barriers to sharing so that they may be addressed in future research.

## Open practices statement

All data exclusions and measures conducted during this study are reported. All data, materials, and analysis code pertaining to this study have been made publicly available on the Open Science Framework (https://osf.io/64qvb/). To facilitate reproducibility this manuscript was written by interleaving regular prose and analysis code (https://osf.io/7syrt/) using knitr and papaja, and is available in a Code Ocean container (https://doi.org/10.24433/CO.241ffbb1-5b81-44bd-94f4-d066b62c5f7f.v2) which re-creates the software environment in which the original analyses were performed.

## Materials and methods

### Sample

Web of Science Essential Science Indicators was used to identify the top 200 most highly-cited articles in the research category “psychology/psychiatry” between 2006 and 2016 (date of search: 31st May, 2017). From these 200 we selected only research articles that would in principle have recorded individual-participant level data, leaving 48 eligible (24 from psychology and 24 from psychiatry). As none of these articles were published after 2011 they may be unrepresentative of recent data sharing trends [3]. To address this, we conducted an additional focused search to identify the top 200 most highly articles published during the 2014–2016 time period. Applying the same eligibility criteria, we identified 63 eligible studies (17 from psychology and 46 from psychiatry). Combining the two groups of studies resulted in a total sample size of 111 articles, 70 of which were from psychiatry and 41 of which were from psychology.

Essential Science Indicators include the top 1% most-cited papers after accounting for field and year of publication. At the time of the search, they contained 1803 psychology and psychiatry papers published in 2006–2011 and 1285 published in 2014–2016. Therefore, our sample includes papers in the top 0.11% and top 0.16% of citations for 2006–2011 and 2014–2016, respectively. The median number of citations for eligible articles was 64 in psychiatry (range: 45–1390) and 470 in psychology (range: 48–1768).

Based on examination of article titles and abstracts, the types of eligible studies included field experiments or surveys (n = 26), epidemiological surveys (n = 23), randomized clinical trials (n = 23), case-control studies (n = 16), laboratory experiments (n = 13), and development of stimuli, surveys, or diagnostic screening instruments (n = 10). A data file containing all 400 sampled studies is available here: https://osf.io/84cm5/

### Procedure

The 111 corresponding authors of eligible articles were contacted on 23rd June 2017 using the e-mail address provided in the published article. Two reminder e-mails were sent to non-respondents at 2 weeks intervals as required. If e-mails bounced, and before sending any reminders, we performed an Internet search to obtain the most up-to-date contact details for the corresponding author. If we could not identify an active e-mail address, we attempted to contact a different member of the research team.

The initial contact e-mail (full version available here: https://osf.io/e8gxq/) outlined our goal to promote the availability and usability of raw data from the most influential investigations in psychology and psychiatry by uploading data to an online repository that would ensure their persistence and accessibility. We specifically requested *raw*, *participant-level data that supports all findings [reported in their article]* and mentioned that it would be highly desirable if additional documentation and/or analysis scripts were also be made available. We stressed that contributing researchers could specify access restrictions for the data if they desired. We also highlighted that we were aware that data sharing may not be feasible in all cases and asked that if this were the case, researchers send us their reasons for not sharing. The e-mail concluded by asking researchers to send us their data files (if applicable) and specify their desired level of sharing from one of four options: (1) Online data repository, completely open access; (2) Online data repository, controlled access (please state what prerequisites, criteria and processes you might consider for deciding whether to share the data with others); (3) Sharing with the METRICS team only, not to be shared with anyone else; (4) Cannot share data (please state the key reason(s)).
